# Effect of progesterone administration in male and female smokers on nicotine withdrawal and neural response to smoking cues: role of progesterone conversion to allopregnanolone

**DOI:** 10.1186/s13293-022-00472-w

**Published:** 2022-10-23

**Authors:** Andrew M. Novick, Korrina A. Duffy, Rachel L. Johnson, Mary D. Sammel, Wen Cao, Andrew A. Strasser, Mehmet Sofuoglu, Alexandra Kuzma, James Loughead, A. Leslie Morrow, C. Neill Epperson

**Affiliations:** 1 Department of Psychiatry, School of Medicine, University of CO-Anschutz Medical Campus, 13001 E 17th Pl, Aurora, CO 80045 USA; 2 Department of Biostatistics and Informatics, Colorado School of Public Health, University of CO-Anschutz Medical Campus, Aurora, CO 80045 USA; 3 Department of Psychiatry, Perelman School of Medicine, University of Pennsylvania, Philadelphia, PA 19104 USA; 4 Department of Psychiatry, Yale School of Medicine, Yale University, New Haven, CT 06511 USA; 5 Larner College of Medicine, University of Vermont, Burlington, VM 05405 USA; 6 Departments of Psychiatry and Pharmacology and the Bowles Center for Alcohol Studies, University of North Carolina School of Medicine, Chapel Hill, NC 27514 USA; 7 Department of Family Medicine, School of Medicine, University of CO-Anschutz Medical Campus, Aurora, CO 80045 USA

**Keywords:** Smoking, Progesterone, Allopregnanolone, Sex-differences, Neurosteroids

## Abstract

**Background:**

Progesterone administration has therapeutic effects in tobacco use disorder (TUD), with females benefiting more than males. Conversion of progesterone to the neurosteroid allopregnanolone is hypothesized to partly underlie the therapeutic effects of progesterone; however, this has not been investigated clinically.

**Methods:**

Smokers (*n* = 18 males, *n* = 21 females) participated in a randomized, double-blind, placebo-controlled crossover study of 200 mg progesterone daily across 4 days of abstinence. The ratio of allopregnanolone:progesterone was analyzed in relationship to nicotine withdrawal, smoking urges, mood states, subjective nicotine effects, and neural response to smoking cues.

**Results:**

Allopregnanolone:progesterone ratio interacted with sex to predict withdrawal symptoms (*p* = 0.047), such that females with higher allopregnanolone:progesterone ratios reported lower withdrawal severity (*b* = − 0.98 [− 1.95, − 0.01]; *p* = 0.048). In addition, allopregnanolone:progesterone ratio interacted with sex to predict confusion (*p* = 0.014) and fatigue (*p* = 0.034), such that females with higher allopregnanolone:progesterone ratios reported less confusion (*b* = − 0.45 [− 0.78, − 0.12]; *p* = 0.008) and marginally lower fatigue (*b* = − 0.50 [− 1.03, 0.02]; *p* = 0.062. Irrespective of sex, higher ratios of allopregnanolone:progesterone were associated with stronger “good effects” of nicotine (*b* = 8.39 [2.58, 14.20]); *p* = 0.005) and weaker “bad effects” of nicotine (*b* = − 7.13 [− 13.53, − 0.73]; *p* = 0.029).

**Conclusions:**

Conversion of progesterone to allopregnanolone correlated with smoking-related outcomes in both sex-dependent and sex-independent ways. Sex-dependent effects suggest that conversion of progesterone to allopregnanolone may contribute to greater therapeutic benefits in females but not males with TUD.

*Trial registration* Clinicaltrials.gov registration, retrospectively registered: NCT01954966; https://clinicaltrials.gov/ct2/show/NCT01954966\

**Supplementary Information:**

The online version contains supplementary material available at 10.1186/s13293-022-00472-w.

## Highlights

- Progesterone has sex-dependent effects on smoking measures in individuals with Tobacco Use Disorder.
- Higher conversion of progesterone to allopregnanolone (as indicated by the allopregnanolone:progesterone ratio) was associated with lower nicotine withdrawal in female but not male smokers during a brief abstinence.
- Higher conversion of progesterone to allopregnanolone was associated with lower ratings of confusion and marginally lower ratings of fatigue in female but not male smokers.
- Irrespective of sex, higher conversion of progesterone to allopregnanolone was associated with stronger “good effects” and weaker “bad effects” of nicotine during active smoking.

**Supplementary Information:**

The online version contains supplementary material available at 10.1186/s13293-022-00472-w.

## Introduction

Sex differences in substance use disorders (SUDs) are common and vary depending upon the substance in question. While prevalence for most SUDs may be more common among males [1], tobacco use disorder (TUD) is particularly concerning for females because they become dependent more quickly after exposure [2, 3], experience greater health consequences [4], are less responsive to nicotine replacement therapy [5], and are more likely to relapse after abstinence [6]. While the physiologic underpinnings for these observed sex differences are unknown, growing evidence suggests that neuroactive steroids should be considered given that they act on the brain structures [7–9] and neurotransmitters [7, 10] impacted by nicotine yet differ in level and cyclicity between males and females.

Progesterone (PROG) and its gamma-aminobutyric acid (GABA)-A receptor modulating metabolite, allopregnanolone (ALLO), have been implicated in the pathophysiology and treatment of TUD as well as sex differences in TUD [11, 12]. ALLO modulates processes relevant to TUD, including reward [13–15], mood [16], anxiety [16], and cognition [17, 18]. Delta-subunit containing GABA-A receptors for ALLO are located in similar brain regions as nicotinic acetylcholine receptors [19, 20], suggesting ALLO’s capacity to influence nicotine’s effects. A recent study in male rats found that a positive allosteric modulator of delta-subunit containing GABA-A receptors in the amygdala blocked stress-induced enhancement of nicotine self-administration [21]. Unlike the GABA-A receptor, there is a lack of evidence for a direct role of the PROG receptor in processes related to TUD; however, in vitro experiments demonstrate that PROG and ALLO both separately inhibit activity of nicotinic acetylcholine receptors [22, 23].

A clinical rationale for the therapeutic effects of PROG and ALLO in TUD developed from studies of female smokers who demonstrated greater success at quitting during the luteal phase of the menstrual cycle when endogenous levels of PROG and ALLO are both high relative to the follicular phase [24, 25]. Furthermore, women demonstrate lower activation of reward-related brain regions, specifically the putamen, when viewing smoking cues in the luteal versus follicular phase of the menstrual cycle [26].

Among smokers, PROG administration decreases cigarette cravings [27–30], decreases amount smoked [29, 31], decreases the rewarding effects of nicotine [29, 30, 32], and increases time to relapse [33]. Females may benefit from PROG administration more than males [30, 33, 34]. In studies examining sex differences in response to PROG administration, females but not males showed improved cognitive function during acute smoking abstinence [30] and increased time to smoking relapse [33]. In line with these findings, we found that PROG administration relative to placebo increased withdrawal severity in males but not females [34]. Similarly, studies in humans and rodents with cocaine dependence demonstrate a benefit of PROG administration in females but not males [35–38], but see [17].

Limited research suggests that the primary effect of PROG on TUD may be through its conversion to ALLO—but whether this applies to both males and females in humans remains unclear. In male mice, administration of PROG or ALLO decreases anxiety-like behavior during nicotine withdrawal, but the effect of PROG on anxiety-like behavior is blocked when conversion to ALLO is inhibited [39], suggesting that PROG’s effects act through its conversion to ALLO. A study conducted on patients with cocaine use disorder found that high levels of ALLO following administration of 400 mg PROG (or placebo) was associated with improved mood and cognitive performance under stress as well as reduced cocaine cravings; however, sex differences were not investigated [17]. Sex differences in GABA-A receptor density [40, 41] suggest that males and females with TUD might have differential responses to PROG depending on the extent to which it is converted to ALLO.

Teasing out the effect of PROG versus ALLO on smoking behavior in humans is complex yet important as PROG is easily administered and well-tolerated [28–30, 32–34, 38]. Given evidence that PROG conversion to ALLO is necessary to observe behavioral and neural effects relevant to its use as a potential treatment for some SUDs, we measured ALLO:PROG ratio at multiple timepoints in male and female smokers receiving PROG and placebo during brief abstinence in a double-blind, randomized crossover study. Then, we tested whether ALLO:PROG ratio interacted with sex to predict therapeutic benefits across a comprehensive set of smoking-related behavioral, psychological, and neural measures.

## Methods and materials

### Participants

We utilized data collected in a double-blind, randomized, placebo-controlled crossover study [34]. Briefly, non-treatment-seeking male and female daily smokers 18–50 years old without current psychiatric or major medical illnesses were recruited for the study. Participants smoked at least 10 cigarettes per day over the past year, with a score of at least three on the Fagerström Test for Nicotine Dependence (FTND), and have an expired carbon monoxide (CO) level of at least 11 ppm. A full list of inclusion/exclusion criteria can be found in Table 1. Further information on study screening is provided in Additional file 1. Of 131 potential participants screened for the study, 81 were enrolled, 66 were randomized, and 52 completed both periods. Of the 66 randomized, the analyzed sample had 39 participants for whom neurosteroid data were available. The Supplemental Materials contain information on missing data and participant attrition (Additional file 1: Fig S1).

**Table 1:** Inclusion and exclusion criteria for study participation

Inclusion criteria
• Adults 18–50 years old
• Daily smokers for ≥ 12 months who smoke ≥ 10 cigarettes/day
• Fagerström test for nicotine dependence ≥ 3 • CO levels ≥ 11
• Not currently seeking treatment for nicotine dependence • Clear urine drug screen (marijuana permissible)
• Not currently pregnant (females only) • Regular menstrual cycle (females only)
Exclusion criteria
• Current major medical illness
• Lifetime history of psychotic disorder (DSM-IV)
• Other Axis 1 psychiatric disorder within past year (DSM-IV)
• Lifetime history of substance dependence disorder other than nicotine (DSM-IV)
• History of substance use disorder other than nicotine within past 2 years (DSM-IV)
• Heavy alcohol use within past year (> 7 drinks/week or > 3 drinks/occasion for females; > 14 drinks/week or > 4 drinks/occasion for males)
• Current regular use of any tobacco products other than cigarettes
• Current regular use of psychotropic medication
• Contraindications to progesterone use (e.g., thrombophlebitis, stroke)
• Known progesterone or peanut allergy (Prometrium^®^ contains peanut oil)
• Left-handed (for consistency in neuroimaging) Weighs ≥ 300 lbs. (contraindication for fMRI)
• Metallic implants (contraindication for fMRI)
• Claustrophobic (contraindication for fMRI)

### Study design

**Fig. 1:**
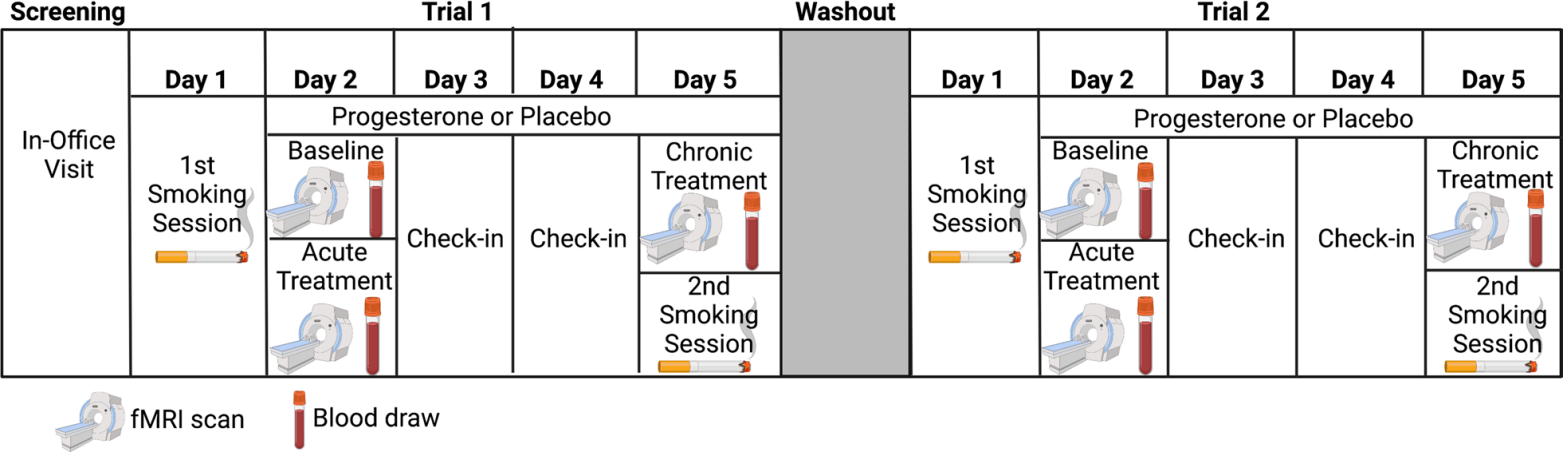
Crossover study design. Functional magnetic resonance imaging (fMRI) scans measured neural activation in response to smoking and neutral cues. Participants completed measures of withdrawal symptoms, smoking urges, and mood states before each brain scan. Blood draws measured levels of neurosteroids, such as progesterone and allopregnanolone, after each brain scan. Smoking sessions measured change in carbon monoxide (CO) levels, number of cigarettes smoked, inhalation volume for the first cigarette, total inhalation volume during the session, and subjective nicotine effects at baseline and after a 4-day abstinence

As described in Novick et al. [34], participants received oral micronized PROG (200 mg daily of Prometrium^®^) or placebo over a 4-day abstinence period followed by a washout period and subsequent crossover to the other period (for an overview of the study procedure and design, see Fig. 1). On day 1, participants received no drug but completed a 2-h ad lib smoking session to measure baseline smoking behavior and subjective responses to nicotine. They continued smoking as usual following the session. On day 2, participants smoked their last cigarette prior to entering the testing facility, beginning the abstinence period. They underwent the baseline fMRI scan within 30 min of their last cigarette. After the baseline scan, they were administered 200 mg PROG or placebo, and then they underwent a second fMRI scan to measure acute effects 3 h later (acute treatment timepoint). On days 3 and 4, participants self-administered 200 mg progesterone or placebo. On day 5, participants self-administered their final dose of progesterone or placebo and underwent a third fMRI scan to measure chronic effects (chronic treatment timepoint). This was followed by a post-treatment 2-h ad lib smoking session. Before each fMRI scan, participants completed questionnaires to measure withdrawal symptoms, smoking urges, and mood states. Each fMRI measured neural activation while participants viewed smoking and neutral cues. After each fMRI scan, blood was drawn to measure circulating levels of neurosteroids (e.g., PROG and ALLO).

After completion of the first period, male participants had a 1- to 2-week washout period before crossover to the second period in which the above procedures were repeated. All menstruating female participants had a 1- to 3-month washout period before crossover. For females who were naturally cycling or were continuing to experience menstrual cycles while using a long-acting form of contraception (e.g., an intrauterine device), all study days took place within 7 days of self-reported menses onset (early follicular phase). Menstruating females taking oral contraceptive pills were scheduled during the contraceptive placebo pill days of their cycle. This ensured that progesterone was being administered in the context of low endogenous estradiol and progesterone to minimize interactions with exogenous progesterone, a commonly used approach in progesterone trials [29, 30, 32, 34].

### Procedures

#### Progesterone administration

Micronized PROG (Prometrium) 200 mg capsules were obtained from Catalent Pharma Solutions, St. Petersburg, FL. This compound is identical in chemical structure to endogenous PROG synthesized in the body [42]. Similar placebo capsules were prepared by the Investigational Drug Service at the University of Pennsylvania. The 200 mg once daily dose is commonly used to prevent endometrial hyperplasia in postmenopausal women receiving estrogen replacement therapy [42] and is effective in attenuating smoking cravings and positive effects of smoking in females after overnight abstinence [29].

#### Contingency management

Contingency management was used to reinforce abstinence with modest monetary payments [43]. To assess compliance with the abstinence protocol, study staff measured the participant’s alveolar CO levels twice per day on days 3 and 4 and once on day 5. To be considered compliant, participants needed a CO level of less than 10 ppm [30, 44] or, if greater, it had to be continually decreasing across days 3–5 [34, 45]. Based on CO levels, 11 participants had at least one smoking slip for a total of 16 slips across timepoints—8 in the PROG period and 8 in the placebo period. However, we used an intention-to-treat analytic approach to be conservative in our treatment effect estimates and therefore did not exclude these participants.

#### Outcome measures

##### Behavioral measures

Participants completed a 2-h ad lib smoking session on days 2 and 5. Before and after each smoking session, expired CO levels were measured using the Vitalograph Breathco™ carbon monoxide monitor to gauge acute exposure to tobacco smoke [46]. To assess inhalation volume for the first cigarette and total inhalation volume over the session, participants smoked through a plastic cigarette holder, fitted to the filter end of the cigarette, and connected to a smoking topography device (CreSS from Plowshare Technologies). The number of cigarettes smoked was also measured. After smoking, participants completed the Nicotine Effects Questionnaire (NEQ) to measure the subjective effects of nicotine (see Table 2 for further details).

**Table 2:** Descriptions of self-report measures

Measure	Description
Nicotine Effects Questionnaire	The NEQ is a validated 4-item scale in which participants were asked to rate the strength of nicotine, experience of a head rush, as well as the good and bad effects of nicotine on a 0–100 sliding scale [47]
Nicotine Withdrawal Symptom Checklist	The NWSC (also known as the Minnesota Nicotine Withdrawal Scale) is a validated 8-item scale of withdrawal symptoms, such as irritability, anxiety, insomnia, and depression, rated on a 5-point scale [48]
Questionnaire on Smoking Urges-Brief	The QSU-Brief is a validated 10-item scale in which participants rate the extent to which they agree with statements related to smoking urges and cravings on a 7-point scale [49]
Questionnaire on Smoking Urges	The QSU is a longer, 32-item version of the QSU-Brief in which participants rate items on a 7-point scale [50]
Profile of Mood States	The POMS is a 65-item questionnaire that measures six mood states: tension–anxiety, depression–dejection, anger–hostility, confusion–bewilderment, fatigue, and vigor [51]

##### Psychological measures

Participants completed the following self-report measures: the Nicotine Withdrawal Symptom Checklist (NWSC, also known as the Minnesota Nicotine Withdrawal Scale), the Brief Questionnaire of Smoking Urges (QSU-Brief), the Questionnaire on Smoking Urges (QSU), and the Profile of Mood States (POMS) (see Table 2 for further details). The NWSC, QSU-Brief, and POMS were administered at the baseline, acute, and chronic treatment timepoints. The QSU was administered only at the chronic treatment timepoint.

##### Neural measures

**Fig. 2:**
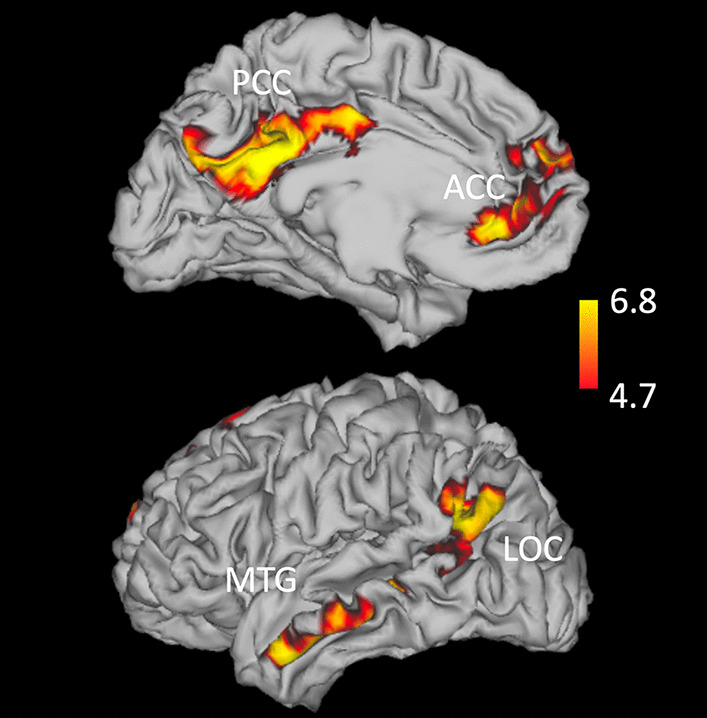
*Z*-score map of functionally defined regions of interest (ROIs) for the contrast smoking cue minus neutral measured at the baseline session. ROI masks were created via voxel-wise correction at *p* = 0.05. Percent signal change was extracted from these ROIs for all 6 sessions for subsequent analysis. Specific details on ROIs, including voxels, coordinates, and specific *z*-score can be found in Additional file 1: Table S13

Neural activation in response to smoking cues was assessed with BOLD imaging using a validated task [52, 53] described previously [34] in which participants viewed grayscale images of smoking and neutral cues. Smoking cues were images of people smoking cigarettes, holding cigarettes, and handling smoking-related items, such as lighters. Neutral cues were images matched for visual content (e.g., a person with a pen in their mouth). To ensure participant engagement, a target stimulus (picture of an animal) was presented infrequently, and participants were instructed to respond with a button press. The task consisted of 20 smoking, 20 neutral, and four target images, with each image presented for four seconds. During the interstimulus interval, a fixation point appeared on a grey screen for a variable length of time (between 6–14 s). Midway through the task, the fixation point appeared during a 24-s rest period. Stimuli class was pseudo-randomized with no more than two images of a given image type being presented consecutively. The total task duration was 10 min and 36 s. Functionally defined regions of interest (ROIs) were derived from whole-brain group analysis of the baseline session smoking cue minus neutral cue contrast using FLAME (FMRIB's Local Analysis of Mixed Effects) [54] (Fig. 2). ROI masks were created via voxel-wise correction at *p* = 0.05. Percent signal change was extracted from ROIs that met a threshold of 200 voxels for all 6 sessions and 3 contrasts (smoking, neutral, smoking minus neutral). Additional information on fMRI acquisition and processing can be found in Additional file 1.

#### Neurosteroid measures

After each fMRI scan, a blood sample was taken to measure the following neurosteroids: PROG, ALLO, 3a,5b-THP, pregnenolone, 3a,5a-androsterone, 3a,5b-androsterone, 3a,5a-androstandiol, 3a,5b-androstandiol, 3a,5a-THDOC, and 3a,5b-THDOC. Neurosteroids were analyzed using gas chromatography–mass spectrometry, as described previously [55, 56]. See Additional file 1: Fig S2 for a diagram of the PROG metabolic pathway depicting the neurosteroids that were measured.

### Statistical plan

To test whether individual differences in the conversion of PROG to its metabolite ALLO predicted our behavioral, psychological, and neural smoking measures, we calculated the ratio of ALLO to PROG (ALLO:PROG ratio) at three timepoints: baseline, the acute timepoint, and the chronic timepoint. Ratios were log-transformed to normalize their distribution. Because our previous paper reported on the results of our clinical trial [34] and we were interested in the association between ALLO:PROG ratio in the blood and smoking-related psychological, behavioral, and neural outcomes, we collapsed data across the PROG and placebo periods to increase statistical power. For all analyses, we controlled for drug condition. Although the main the purpose of this manuscript was to investigate associations with ALLO:PROG ratio, we present analyses with PROG levels and ALLO levels separately in Additional file 1.

Given our prediction that sex would moderate the association between ALLO:PROG ratio and our outcomes, all models first tested for an interaction between sex and ALLO:PROG ratio. Then, if the Type III interaction *p*-value was not significant at the level $\alpha$ = 0.05, we tested for main effects of sex and ALLO:PROG ratio within the same model. We conducted these analyses for each of our psychological, behavioral, and neural smoking measures, modeling repeated outcome measures using generalized estimating equations (GEEs).

Our models differed in time point structures because different outcomes were measured at different timepoints depending on clinical interest. Our models assessing psychological outcomes used three repeated measures (baseline, acute, and chronic) per period for NWSC, QSU-Brief, and POMS; however, the QSU was only measured at the chronic timepoint for each period. Our behavioral outcomes (change in CO levels, number of cigarettes smoked, inhalation volume for first cigarette, total inhalation volume during the session, and subjective nicotine effects) were assessed at the final smoking session for each period. Our models assessing neural outcomes used two repeated measures (acute minus baseline, chronic minus baseline) to measure change in neural activation from baseline for each period. Neural activation outcomes were calculated as the difference between neural activation in response to the smoking cues minus the neutral cues for each period. This was computed separately for all four regions of interest (ROIs) identified using whole brain analysis: the posterior cingulate cortex (PCC), anterior cingulate cortex (ACC), left lateral occipital cortex, and left middle temporal gyrus. To account for repeated measures, models included an exchangeable correlation structure and adjusted for time. All models adjusted for drug condition as well as randomization order to account for potential order effects due to the crossover design. Analyses were performed in SAS 9.4 (SAS Institute, Cary, NC).

## Results

### Participant demographics

Thirty-nine participants (age: M = 36.1; SD = 8.7) were randomized to condition order (either placebo first or PROG first). Male (*n* = 18) and female (*n* = 21) daily smokers were represented in similar numbers in the overall sample. See Additional file 1: Table S1 for additional demographic information on our sample.

### Levels of progesterone, allopregnanolone, and allopregnanolone-to-progesterone ratio

In Table 3a–c, we summarize the blood levels of PROG and ALLO measured at baseline and after participants received acute and chronic doses of oral micronized PROG/placebo.

**Table 3:** a–c Raw data on blood levels of progesterone and allopregnanolone

	Placebo				Progesterone		
	*N*		Mean (SD)	Median [min, max]	*N*	Mean (SD)	Median [min, max]
**Baseline**							
**a. Progesterone (pg/mL)**							
Males		15	361.2 (120.3)	347.0 [200, 580]	18	362.3 (144.1)	339.5 [175, 733]
Females	16		837.1 (869.1)	459.0 [109, 3070]	17	932.3 (1457.4)	405.0 [153, 6090]
*Acute*							
Males	14		311.1 (104.3)	302.0 [200, 590]	17	5264.1 (3317.8)	4690.0 [900, 14030]
Females	15		959.1 (1054.8)	439.0 [149, 3520]	16	10,369.1 (9925.7)	8440.0 [305, 36500]
*Chronic*							
Males	15		285.9 (104.3)	272.0 [198, 529]	17	8199.4 (6521.2)	5630.0 [1730, 20700]
Females	15		1444.8 (2237.1)	406.0 [132, 8230]	15	10,732.0 (10,218.6)	9730.0 [1540, 37360]
**b. Allopregnanolone (pg/mL)**							
*Baseline*							
Males	15		54.4 (76.1)	26.5 [3.0, 312.5]	18	53.6 (75.2)	31.2 [3.5, 286.2]
Females	14		101.7 (58.6)	94.8 [43.2, 250.6]	17	122.3 (85.2)	98.3 [10.7, 319.7]
*Acute*							
Males	17		70.1 (72.3)	41.1 [9.4, 258.3]	19	6893.4 (7036.8)	4548.7 [951.2, 31,885.8]
Females	17		104.2 (68.5)	103.5 [22.1, 246.6]	15	5430.5 (4632.8)	3905.6 [115.0, 12,727.3]
*Chronic*							
Males	17		56.5 (60.7)	36.3 [5.1, 252.5]	19	6474.4 (4793.7)	5600.4 [1007.4, 19,775.7]
Females	15		142.2 (92.2)	121.4 [32.0, 339.1]	16	5551.5 (4477.0)	5350.3 [213.2, 17,084.7]
**c. Log (allopregnanolone/progesterone)**							
*Baseline*							
Males	14		− 2.3 (0.9)	− 2.3 [− 4.4, − 0.6]	17	− 2.5 (1.0)	− 2.2 [− 4.6, − 0.8]
Females	13		− 1.7 (1.1)	− 1.4 [− 3.6, − 0.5]	17	− 1.7 (0.9)	− 1.8 [− 3.1, − 0.1]
*Acute*							
Males	13		− 1.9 (0.9)	− 1.9 [− 3.5, 0.1]	17	0.0 (0.5)	0.1 [− 1.4, 0.8]
Females	14		− 1.8 (1.1)	− 1.5 [− 3.7, − 0.4]	13	− 0.6 (0.7)	− 0.8 [− 1.5, 0.8]
*Chronic*							
Males	15		− 1.9 (0.8)	− 1.9 [− 3.7, − 0.2]	17	− 0.1 (0.8)	0.0 [− 1.5, 0.8]
Females	15		− 1.7 (1.3)	− 1.0 [− 4.2, − 0.2]	15	− 0.8 (1.0)	− 0.7 [− 3.3, 1.2]

Mean, standard deviation, median, and range of (a) progesterone, (b) allopregnanolone, and (c) the ratio of allopregnanolone:progesterone at baseline, the acute treatment timepoint, and the chronic treatment timepoint separated by drug (progesterone vs. placebo) and stratified by sex

### Psychological measures

**Fig. 3:**
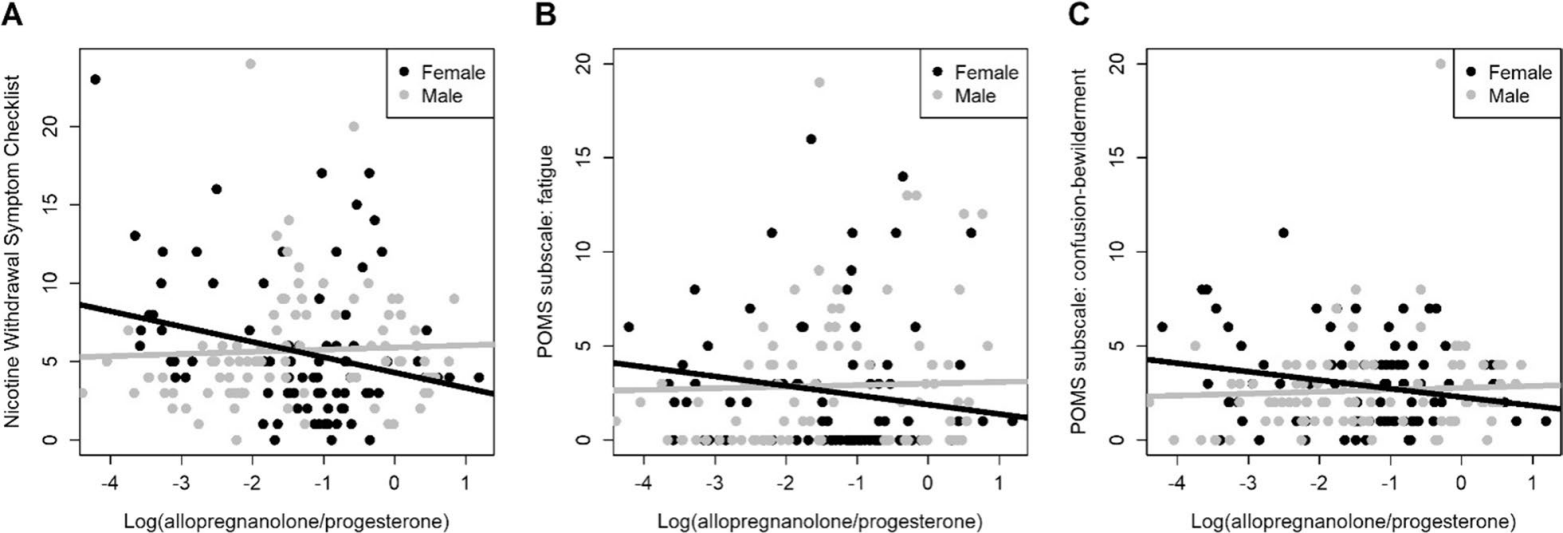
Significant interaction of allopregnanolone:progesterone ratio and sex on withdrawal symptoms and mood states. **A** Allopregnanolone:progesterone ratio and sex significantly interacted to predict nicotine withdrawal symptoms (*p* = 0.047) on the Nicotine Withdrawal Symptom Checklist. The simple slope for females was significant, such that having a higher ratio of allopregnanolone:progesterone was associated with lower withdrawal symptoms (*p* = 0.048). **B** Allopregnanolone:progesterone ratio significantly interacted with sex to predict fatigue on the Profile of Moods Scale (POMS) (*p* = 0.034). For females, a higher ratio of allopregnanolone:progesterone was marginally associated with lower reports of fatigue (*p* = 0.062). **C** Allopregnanolone:progesterone ratio significantly interacted with sex to predict confusion on the POMS. For females, higher ratios of allopregnanolone:progesterone were associated with lower reports of confusion (*p* = 0.008). For details see Table 4

Sex interacted with ALLO:PROG ratio to predict withdrawal severity (NWSC: difference in slopes: *b* = − 1.12 [− 2.01, − 0.22]; *p* = 0.047) but not smoking urges (QSU-Brief: *p* = 0.420; QSU: *p* = 0.179). For NWSC, the simple slope for females showed lower nicotine withdrawal symptoms with higher ratios of ALLO:PROG (*b* = − 0.98 [− 1.95, − 0.01]; *p* = 0.048; Fig. 3). For the mood state subscales (POMS), the interaction effect was significant for fatigue (difference in slopes: *b* = − 0.58 [− 1.08, − 0.08]; *p* = 0.034; Fig. 3) and for confusion–bewilderment (difference in slopes: *b* = − 0.55 [− 0.99, − 0.11]; *p* = 0.014; Fig. 3). The simple slope was significant for confusion–bewilderment for females such that higher ALLO:PROG ratio was associated with reduced confusion–bewilderment (*b* = − 0.45 [− 0.78, − 0.12]; *p* = 0.008; Fig. 3). For all interaction model results, see Table 4. Given that the interaction effect and simple slopes by sex were not significant for QSU-Brief, QSU, and other five POMS subscales, we tested for main effects of ALLO:PROG ratio and sex. However, no significant main effects emerged for any of the measures (*p*s > 0.05). For all main effect model results, see Table 5.

**Table 4:** Interaction effect of allopregnanolone:progesterone ratio and sex on self-reported psychological measures

Outcome	Log (allo/prog) by sex interaction *p* value	Within females: Effect of log(allo/prog) (95% CI)	*p* value	Within males: Effect of log(allo/prog) (95% CI)	*p* value	Difference between slopes (95% CI)
QSU-Brief	0.420	− 2.05 (− 5.36, 1.26)	0.225	− 0.40 (− 2.92, 2.12)	0.756	− 1.65 (− 5.46, 2.16)
NWSC	0.047^b^	− 0.98 (− 1.95, − 0.01)	0.048^b^	0.13 (− 0.39, 0.67)	0.612	− 1.12 (− 2.01, − 0.22)
QSU^a^	0.179	− 1.07 (− 9.16, 7.03)	0.796	8.81 (− 4.52, 22.15)	0.195	− 9.88 (− 24.29, 4.53)
POMS: tension–anxiety	0.201	− 0.38 (− 1.13, 0.38)	0.330	0.20 (− 0.29, 0.68)	0.428	− 0.57 (− 1.37, 0.22)
POMS: depression–dejection	0.937	0.09 (− 0.57, 0.75)	0.785	0.12 (− 0.12, 0.36)	0.328	− 0.03 (− 0.66, 0.61)
POMS: anger–hostility	0.118	− 0.52 (− 1.24, 0.20)	0.115	0.05 (− 0.40, 0.51)	0.815	− 0.57 (− 1.17, 0.02)
POMS: fatigue	0.034^b^	− 0.50 (− 1.03, 0.02)	0.062	0.08 (− 0.37, 0.53)	0.724	− 0.58 (− 1.08, − 0.08)
POMS: vigor	0.262	1.05 (− 0.16, 2.26)	0.089	0.19 (− 0.69, 1.06)	0.675	0.87 (− 0.55, 2.28)
POMS: confusion–bewilderment	0.014^b^	− 0.45 (− 0.78, − 0.12)	0.008^b^	0.10 (− 0.11, 0.31)	0.352	− 0.55 (− 0.99, − 0.11)

For each outcome, the interaction effect and simple slopes by sex are depicted

*QSU-Brief* Brief Questionnaire on Smoking Urges, *NWSC* Nicotine Withdrawal Symptom Checklist, *QSU* Tiffany Questionnaire of Smoking Urges, *POMS* Profile of Mood States

^a^Only at final scan (‘chronic’ visit); no repeated measures

^b^Statistical significance at level *p* < 0.05

**Table 5:** Main effects of allopregnanolone:progesterone ratio and sex on self-reported psychological measures that did not show a significant interaction effect in Table 4

Outcome	Effect of log(allo/prog)	*p* value	Effect of sex gender [ref: female] (95% CI)	*p* value
QSU-Brief	− 0.94 (− 3.19, 1.31)	0.412	− 0.38 (− 7.17, 6.40)	0.912
QSU^a^	4.38 (− 5.30, 14.06)	0.375	− 17.42 (− 38.37, 3.54)	0.103
POMS: tension–anxiety	0.00 (− 0.46, 0.47)	0.987	0.44 (− 0.92, 1.80)	0.526
POMS: depression–dejection	0.11 (− 0.20, 0.42)	0.490	− 0.26 (− 1.51, 0.98)	0.681
POMS: anger–hostility	− 0.14 (− 0.65, 0.38)	0.606	− 0.29 (− 1.67, 1.08)	0.676
POMS: vigor	0.44 (− 0.32, 1.20)	0.258	2.13 (− 1.55, 5.82)	0.257

*QSU-Brief* Brief Questionnaire on Smoking Urges, *NWSC* Nicotine Withdrawal Symptom Checklist, *QSU* Tiffany Questionnaire of Smoking Urges, *POMS* Profile of Mood States

^a^Only at final scan (‘chronic’ visit); no repeated measures

### Behavioral measures during a laboratory-based smoking session

Sex did not interact with ALLO:PROG ratio to predict any of the smoking behaviors measured at the end of the 4-day abstinence period: change in CO levels from before and after the smoking session (*p* = 0.237), number of cigarettes smoked (*p* = 0.148), inhalation volume for first cigarette (*p* = 0.803), total inhalation volume during the session (*p* = 0.256), and subjective effects from smoking (all four items on NEQ; *p* > 0.05). However, the simple slope for males was significant for two items on the NEQ. For males, higher ratios of ALLO:PROG were associated with higher perceived good effects (*b* = 9.36 [1.72, 17.00]; *p* = 0.016) and lower perceived bad effects (*b* = − 11.34 [− 20.50, − 2.18]; *p* = 0.015). For all interaction model results, see Table 6. Given the lack of a significant interaction effect, we tested for main effects of ALLO:PROG ratio and sex. The only two significant findings were that higher ratios of ALLO:PROG were associated with stronger “good” (*b* = 8.39 [2.59, 14.20]; *p* = 0.005) and weaker “bad” (*b* = − 7.13 [− 13.53, − 0.73]; *p* = 0.029) effects from smoking, which was driven by males as indicated above. For all main effect model results, see Table 7.

**Table 6:** Interaction effect allopregnanolone:progesterone ratio and sex on final smoking session measures

Outcome at final smoking session	Log(allo/prog) by sex interaction *p* value	Within females: Effect of log(allo/prog) (95% CI)	*p* value	Within males: Effect of log(allo/prog) (95% CI)	*p* value	Difference (95% CI) between slopes
CO (post–pre)	0.237	− 1.81 (− 4.12, 0.51)	0.127	0.55 (− 2.15, 3.26)	0.689	− 2.36 (− 5.92, 1.20)
Number of cigarettes	0.148	− 0.13 (− 0.42, 0.17)	0.401	0.20 (− 0.20, 0.61)	0.327	− 0.33 (− 0.72, 0.06)
Volume smoked (1st cigarette)	0.803	− 77.9 (− 222.6, 88.9)	0.360	− 49.5 (− 286.7, 187.7)	0.683	− 28.3 (− 251.0, 194.3)
Total volume smoked	0.256	193.1 (− 410.6, 796.8)	0.531	548.2 (− 120.4, 1216.7)	0.108	− 355.0 (− 889.9, 179.8)
NEQ1 (feel strength of nicotine)	0.983	2.54 (− 3.22, 8.31)	0.387	2.46 (− 3.86, 8.79)	0.445	0.08 (− 7.18, 7.34)
NEQ2 (feel “good” effects of nicotine)	0.519	6.45 (− 0.71, 13.60)	0.077	9.36 (1.72, 17.00)	0.016^a^	− 2.91 (− 11.94, 6.12)
NEQ3 (feel “bad” effects of nicotine)	0.140	− 2.99 (− 9.99, 4.01)	0.402	− 11.34 (− 20.50, − 2.18)	0.015^a^	8.35 (− 1.53, 18.23)
NEQ4 (head rush)	0.589	− 8.62 (− 18.82, 1.57)	0.097	− 4.49 (− 16.22, 7.24)	0.453	− 4.14 (− 19.20, 10.93)

For each outcome, the interaction effect and simple slopes by sex are depicted

*NEQ* Nicotine Effects Questionnaire

^a^ Statistical significance at level *p* < 0.05

**Table 7:** Main effects of allopregnanolone:progesterone ratio and sex on smoking session measures that did not show a significant interaction effect in Table 6

Outcome at final smoking session	Effect of log(allo/prog) (95% CI)	*p* value	Effect of sex [ref: female] (95% CI)	*p* value
CO (post–pre)	− 0.65 (− 2.58, 1.27)	0.504	1.18 (− 3.40, 5.75)	0.614
Number of cigarettes	0.04 (− 0.29, 0.37)	0.821	− 0.15 (− 0.87, 0.57)	0.684
Volume smoked (1st cigarette)	− 65.4 (− 232.8, 102.1)	0.444	− 129.1 (− 489.6, 231.5)	0.483
Total volume smoked	369.9 (− 229.0, 968.8)	0.226	− 1030.0 (− 2671.0, 611.1)	0.219
NEQ1 (feel strength of nicotine)	2.81 (− 2.17, 7.79)	0.269	− 0.24 (− 17.84, 17.35)	0.979
NEQ2 (feel “good” effects of nicotine)	8.39 (2.58, 14.20)	0.005^a^	− 12.03 (− 28.98, 4.93)	0.165
NEQ3 (feel “bad” effects of nicotine)	− 7.13 (− 13.53, − 0.73)	0.029^a^	14.80 (− 1.05, 30.64)	0.067
NEQ4 (head rush)	− 5.41 (− 14.59, 1.77)	0.125	16.33 (− 4.43, 37.09)	0.123

*NEQ* Nicotine Effects Questionnaire

^a^Statistical significance at level *p* < 0.05

### Neural activation when viewing smoking cues

No interactions of ALLO:PROG ratio and sex emerged for any of the ROIs tested (see Table 8). Given the lack of significant interactions, we tested for main effects of ALLO:PROG ratio and sex. For all main effect model results, see Table 9.

**Table 8:** Interaction of allopregnanolone:progesterone ratio and sex on neural activation when viewing smoking cues (minus neutral cues)

Outcome	Log(allo/prog) by sex interaction *p* value	Within females: Effect of log(allo/prog) (95% CI)	*p* value	Within males: Effect of log(allo/prog) (95% CI)	*p* value	Difference in slopes (95% CI)
PCC	0.181	0.03 (− 0.07, 0.14)	0.504	− 0.05 (− 0.13, 0.03)	0.253	0.08 (− 0.03, 0.20)
ACC	0.406	− 0.01 (− 0.09, 0.08)	0.904	− 0.04 (− 0.10, 0.01)	0.133	0.04 (− 0.05, 0.13)
Lateral occipital cortex (left)	0.201	0.00 (− 0.07, 0.08)	0.900	− 0.05 (− 0.12, 0.02)	0.172	0.05 (− 0.03, 0.13)
Middle temporal gyrus (left)	0.213	0.04 (− 0.06, 0.13)	0.467	− 0.02 (− 0.09, 0.05)	0.517	0.06 (− 0.03, 0.15)

For each region of interest, the interaction effect and simple slopes by sex are depicted

*PCC* posterior cingulate cortex, *ACC* anterior cingulate cortex

**Table 9:** Main effects allopregnanolone:progesterone ratio and sex on neural activation when viewing smoking cues (minus neutral cues) in regions that did not show a significant interaction effect in Table 8

Outcome	Effect of log(allo/prog)	*p* value	Effect of sex [ref: female] (95% CI)	*p* value
PCC	− 0.02 (− 0.10, 0.06)	0.564	0.14 (0.00, 0.28)	0.048^a^
ACC	− 0.03 (− 0.09, 0.02)	0.243	0.07 (− 0.06, 0.20)	0.292
Lateral occipital cortex (left)	− 0.03 (− 0.10, 0.03)	0.318	0.09 (− 0.02, 0.21)	0.122
Middle temporal gyrus (left)	− 0.01 (− 0.07, 0.06)	0.837	0.09 (− 0.03, 0.21)	0.160

*PCC* posterior cingulate cortex, *ACC* anterior cingulate cortex

^a^Statistical significance at level *p* < 0.05

## Discussion

To our knowledge, this is the first study to assess the extent to which conversion to ALLO following PROG or placebo treatment is associated with smoking-related behavioral, psychological, and neural outcomes. Significant sex differences emerged in how ALLO:PROG ratios correlated with various smoking-related outcomes. In females, but not males, greater ALLO:PROG ratios were associated with lower nicotine withdrawal, lower confusion, and marginally lower fatigue. Our findings add to previous studies demonstrating differential effects of PROG on smoking outcomes depending on sex [30, 33, 34] and suggest that the conversion of PROG to ALLO produces different effects in males and females. More specifically, some of our findings suggest that females but not males benefit from higher levels of ALLO relative to PROG levels and that conversion of PROG to ALLO may underlie aspects of its therapeutic effects in substance use disorders in females. Interestingly, despite these correlations, we did not see these correlations with ALLO levels alone (Additional file 1). This may indicate differential and perhaps opposing effects of ALLO and PROG. For example, ALLO has GABAergic effects while PROG does not, and PROG has genomic actions whereas ALLO does not [57].

Previously, we reported that, relative to placebo, PROG treatment resulted in increased withdrawal symptoms in males undergoing brief abstinence but had no effect in females [34]. The present results reveal that a higher ALLO:PROG ratio in females is associated with decreased withdrawal symptoms, raising the possibility that PROG treatment in females might be even more effective in relieving withdrawal symptoms if ALLO conversion could be increased. Furthermore, although ALLO:PROG ratio did not show a significant relationship to withdrawal symptoms in males, its simple slope was in the opposite direction to that in females, suggesting that increases in conversion of ALLO to PROG could lack a therapeutic benefit in males on nicotine withdrawal and may explain why in our previously published clinical trial analyses males showed worse withdrawal symptoms during abstinence with PROG treatment compared with placebo [34].

Higher ALLO:PROG ratios predicted less confusion in females but not males. Although subjective reports of confusion differ from objective measures of cognitive performance, another study found that exogenous PROG improved performance on the Stroop task in female but not male abstinent smokers [30]. Attentional difficulties are commonly encountered during abstinence from smoking and alleviation of such cognitive difficulties has been proposed as a treatment strategy for TUD [58–60]. An additional sex by ALLO:PROG ratio interaction emerged for reported fatigue, with females reporting marginally less fatigue with greater ALLO:PROG ratio. This has relevance for TUD given that higher levels of exhaustion are associated with greater tendency for relapse [61].

Little research exists to explain why higher ALLO:PROG ratios were associated with more beneficial effects in female versus male smokers. Two studies suggest that female smokers may have a GABAergic deficit that PROG to ALLO conversion could alleviate [62, 63]. Specifically, female chronic smokers have lower levels of ALLO in the blood compared to non-smokers [62], suggesting less basal GABAergic neurosteroid activity. In a separate study that evaluated both males and females, female smokers demonstrated lower brain levels of GABA compared to non-smoking peers, while male smokers did not demonstrate differences in GABA levels compared to non-smoking males [63]. It is also possible that differing steroid metabolism and elimination between sexes could contribute to more therapeutic benefits in females associated with ALLO:PROG ratios. Although there is evidence of similar pharmacokinetics for PROG in males and females [42], if significant sex differences existed for the half-life of ALLO, that might influence the therapeutic impact of PROG conversion to ALLO. However, to evaluate this definitively, it would be necessary to conduct pharmacokinetic studies of both PROG and ALLO and confirm that similar processes were occurring in the brain.

The results did not reveal any correlations between neural activation to smoking cues and the ALLO:PROG ratio in either males in females. This was in line with our previous results that PROG administration (without taking into account hormone levels) lacked significant influence on neural activation to smoking cues compared to placebo. It is important to note that steroid levels were measured in serum and not the brain, which are not always correlated [64, 65]. However, the fact that PROG administration generally increases ALLO:PROG ratio in serum suggests that this likely occurs in the brain as well, given the presence of similar enzymes in the brain and periphery [66]. Nevertheless, smoking-related measures in the current study, including the fMRI measures, might be more tightly linked to brain levels of ALLO or ALLO:PROG ratios since GABA-A receptors are localized to brain regions associated with nicotine action [19, 20].

For outcomes with no interaction of sex by ALLO:PROG ratio, several main effects of ALLO:PROG ratio emerged. Higher ALLO:PROG ratios were associated with more a pleasant smoking experience during the smoking session based on the NEQ. Specifically, higher ALLO:PROG ratios were associated with stronger “good effects” and weaker “bad effects” of smoking. Interestingly, PROG levels demonstrate an opposite effect, at least in females, with higher PROG levels associated with weaker “good effects” of smoking (Additional file 1), suggestive of ALLO and PROG having opposite effects on the smoking experience. The mechanism by which greater ALLO conversion could impact subjective drug effects remains unclear. In male rats, ALLO has been found to increase baseline and morphine-induced dopamine release in the nucleus accumbens [67]. However, a more recent rodent study found that ALLO decreases (rather than increases) electrically evoked burst dopamine release to a greater extent in males versus females, which would be expected to decrease the reported good effects of nicotine [68]. Thus, additional research in humans is needed to understand the mechanisms by which ALLO may impact the subjective effects of nicotine. Nonetheless, these results suggest that the influence of ALLO conversion may not be universally therapeutic.

## Perspectives and significance

The present study provides insight into previously observed sex differences in the therapeutic potential of PROG for treatment of TUD—namely, that females seemed to benefit more than males. The differential relationships between ALLO:PROG ratio and smoking-related outcomes in males and females suggest sex differences in how the brain responds to PROG depending on the extent of metabolic conversion to ALLO. In females, having a higher ALLO:PROG ratio was associated with lower withdrawal symptom severity, lower ratings of confusion, and marginally lower ratings of fatigue during a brief smoking abstinence. Although conversion of PROG to ALLO was associated with more positive subjective effects of smoking for both sexes, in females uncoverted PROG may have an opposite effect and decrease positive subjective effects. To understand the mechanisms driving our effects, future studies should compare the efficacy of PROG administration, direct ALLO administration, and enzyme inhibitors (to prevent conversion to ALLO and other neurosteroid metabolites) on smoking-related measures. In addition, it will be important to determine differences between PROG and various progestins used for contraception on smoking-related measures as the latter are not metabolized into allopregnanolone [69, 70].

## Supplementary Information

**Additional file 1: Fig S1.** CONSORT diagram. **Fig S2.** Progesterone metabolic pathway diagram. The circled neurosteroids were measured in blood samples. **Table S1.** Participant characteristics. **Table S2.** Interaction effect of progesterone levels and sex on self-reported psychological measures. **Table S3.** Main effects of progesterone levels and sex on self-reported psychological measures (for non-significant interactions from Table S2). **Table S3.** Interaction effect of progesterone levels and sex on smoking session measures. **Table S4.** Main effects of progesterone levels and sex on smoking session measures (for non-significant interactions from Table S3). **Table S5.** Interaction of progesterone levels and sex on neural activation when viewing smoking cues minus neutral cues. **Table S6.** Main effects progesterone and sex on neural activation when viewing smoking cues minus neutral cues (for non-significant interactions from Table S5). **Table S7.** Interaction effect of allopregnanolone levels and sex on self-reported psychological measures. **Table S8.** Main effects of allopregnanolone levels and sex on self-reported psychological measures (for non-significant interactions from Table S7). **Table S9.** Interaction effect of allopregnanolone levels and sex on smoking session measures. **Table S10.** Main effects of allopregnanolone levels and sex on smoking session measures (for non-significant interactions from Table S9). **Table S11.** Interaction of allopregnanolone levels and sex on neural activation when viewing smoking cues minus neutral cues. **Table S12.** Main effects allopregnanolone levels and sex on neural activation when viewing smoking cues minus neutral cues (for non-significant interactions from Table S11S). **Table S13.** Functionally defined regions of interest (ROIs) included in analysis, originally identified for the smoking minus neutral contrast at the baseline session with a threshold of 200 voxels and voxel-wise correction of *p* = 0.05.

## Data Availability

The data underlying this article are available through Open Science Framework, at https://dx.doi.org/10.17605/OSF.IO/V7CN8.
