# Supplementary material for: Effect of progesterone administration in male and female smokers on nicotine withdrawal and neural response to smoking cues: role of progesterone conversion to allopregnanolone
Source: Biol Sex Differ. 2022 Oct 23;13:60. doi: 10.1186/s13293-022-00472-w (PMC9590190; doi:10.1186/s13293-022-00472-w)
Supplement: Supplementary file 1 — Additional file 1: Fig S1. CONSORT diagram. Fig S2. Progesterone metabolic pathway diagram. The circled neurosteroids were measured in blood samples. Table S1. Participant characteristics. Table S2. Interaction effect of progesterone levels and sex on self-reported psychological measures. Table S3. Main effects of progesterone levels and sex on self-reported psychological measures (for non-significant interactions from Table S2). Table S3. Interaction effect of progesterone levels and sex on smoking session measures. Table S4. Main effects of progesterone levels and sex on smoking session measures (for non-significant interactions from Table S3). Table S5. Interaction of progesterone levels and sex on neural activation when viewing smoking cues minus neutral cues. Table S6. Main effects progesterone and sex on neural activation when viewing smoking cues minus neutral cues (for non-significant interactions from Table S5). Table S7. Interaction effect of allopregnanolone levels and sex on self-reported psychological measures. Table S8. Main effects of allopregnanolone levels and sex on self-reported psychological measures (for non-significant interactions from Table S7). Table S9. Interaction effect of allopregnanolone levels and sex on smoking session measures. Table S10. Main effects of allopregnanolone levels and sex on smoking session measures (for non-significant interactions from Table S9). Table S11. Interaction of allopregnanolone levels and sex on neural activation when viewing smoking cues minus neutral cues. Table S12. Main effects allopregnanolone levels and sex on neural activation when viewing smoking cues minus neutral cues (for non-significant interactions from Table S11S). Table S13. Functionally defined regions of interest (ROIs) included in analysis, originally identified for the smoking minus neutral contrast at the baseline session with a threshold of 200 voxels and voxel-wise correction of p = 0.05. [file 13293_2022_472_MOESM1_ESM.docx]

**Additional file**

***Study screening***

Potential participants were screened initially over the phone to discuss the study’s purpose, procedures, and general eligibility criteria. For those who met initial eligibility criteria during the phone screening, an electronic medical records review was conducted to check for disqualifying medical conditions and medication usage. Eligible participants were scheduled for a 90-minute in-office evaluation where they gave verbal consent for prescreen procedures. Participants answered screening questions, filled out the FTND (1), completed the Timeline Follow-Back to assess cigarette and alcohol use over the previous 30 days, were administered the Structured Clinical Evaluation for DSM-IV Non-Patient Edition (SCID-IV/NP) by a clinician to assess psychiatric history, had their expired CO measured, and took a urine drug test and pregnancy test (females only).

**fMRI Data Acquisition**

MRI data was acquired with the Siemens Prisma 3T system with a 32-channel transmit/receive head coil (Nova Medical, Wilmington, MA). BOLD fMRI utilized whole-brain, single-shot gradient-echo (GE) echoplanar sequences. Task fMRI was acquired using standard parameters (TR/TE = 1000/30 ms, FOV = 192 mm, matrix = 96x96, slice thickness/gap = 2/0 mm). A 5-min magnetization-prepared, rapid acquisition gradient echo T1-weighted image with integrated motion-correction (TR = 2200 ms, TE = 4.67 ms, TI = 900 ms, FOV = 240 mm, matrix = 256 x 256, effective voxel resolution of 0.9 x 0.9 x 1 mm) was acquired to aid spatial normalization to standard atlas space.

**fMRI Data Processing**

Preprocessing of the fMRI data utilized FEAT (fMRI Expert Analysis Tool) of FSL (FMRIB's Software Library), which included brain extraction with BET (2), slice time correction (interleaved), motion correction using MCFLIRT (3,4) spatial smoothing (6 mm FWHM), and high pass filter (cutoff = 100s). fMRI images were co-registered to the anatomic image using boundary-based registration with integrated distortion correction. The anatomical image was normalized to atlas space (T1 MNI template) using FLIRT (3,4). Preprocessed fMRI data were analyzed using FMRIB's improved general linear model (FILM). FILM prewhitening was applied to the time series to account for temporal autocorrelation (5). The model included explanatory variables (EVs) for smoking cue, neutral, and target stimuli convolved with Double-Gamma Hemodynamic Response Function. Confound EVs, such as temporal derivative and motion parameters (standard + extended), were also included in the model.

**Missing Data**

For any given analysis, randomized participants with data were included even if they were missing some data for that analysis or did not complete both periods. Because participants did not need to have complete data for a given analysis to be included in the sample, generalized estimating equation (GEE) models were used to handle missing data. To be included in analyses, participants were required to have progesterone and allopregnanolone data for at least one time point (39 of 66 participants had this neurosteroid data). Within these 39 participants, data included 212 of 258 possible self-report measures, 209 of 258 possible fMRI scans, and CO levels from 62 of 86 possible smoking sessions. For the neuroimaging analyses, 20 of 66 total participants were missing all neuroimaging data. For 18 participants, this was because they were scanned using a Siemen’s 7T whole body scanner and proton magnetic resonance spectroscopy to measure the neurotransmitter gamma amino-butyric acid (GABA) as registered in clinicaltrials.gov (NCT01954966). However, the 7T was not usable for an extended period and the study switched to using the Siemen’s Prisma 3T system to measure response to smoking and neutral cues using fMRI instead. Two additional participants were not included in the neuroimaging analyses because they missed their initial baseline scan session from which the regions of interest were extracted. For those reasons, 33 participants were included in the neuroimaging analyses.

**Figure S1.** CONSORT diagram.


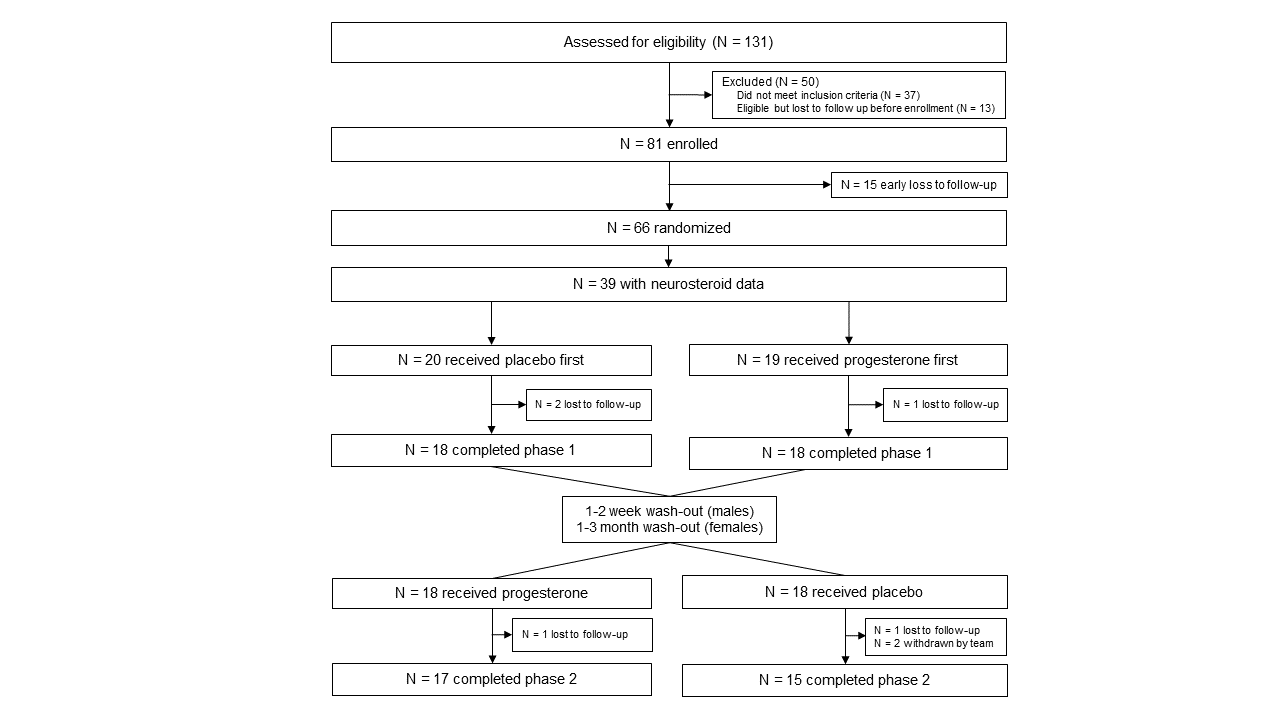


“Completers” as referenced in the manuscript were those who went through both treatment periods but still may have had missing observations. Participants not meeting inclusion criteria (n = 37) were excluded for the following reasons: smoking < 10 cigarettes/day (n = 4), breath CO level < 11 (n = 7), Fagerström Test for Nicotine Dependence < 3 (n = 3), current and/or past exclusionary substance dependence (n = 9), current and/or past exclusionary mental health diagnosis (n = 5), positive drug screen other than cannabis (n = 7), health condition(s) determined from electronic medical records (n = 1), and ≥ 300 pounds (n = 1).

**Figure S2.** Progesterone metabolic pathway diagram. The circled neurosteroids were measured in blood samples.

**Table S1.** **Participant characteristics.** Demographics summarized with means and standard deviations for continuous variables and frequencies and percentages for categorical variables. Participants in the placebo first condition were compared to those in the progesterone first condition for demographic differences, but the only statistically significant difference that emerged was for household income. FTND = Fagerström Test for Nicotine Dependence.

|  | **Overall**  (N = 39) | **Placebo**  **first** (N = 20) | **Progesterone first**  (N = 19) | **P value** |
| --- | --- | --- | --- | --- |
| Age | 36.1 (8.7) | 33.8 (9.8) | 38.5 (6.7) | 0.087 |
| Gender |  |  |  | 0.527 |
| Male | 18 (46.2%) | 8 (40.0%) | 10 (52.6%) |  |
| Female | 21 (53.8%) | 12 (60.0%) | 9 (47.4%) |  |
| Marital status |  |  |  | 0.223 |
| Single | 31 (79.5%) | 17 (85.0%) | 14 (73.7%) |  |
| Married/domestic partner | 3 (7.7%) | 0 (0.0%) | 3 (15.8%) |  |
| Divorced/separated | 5 (12.8%) | 3 (15.0%) | 2 (10.5%) |  |
| Race |  |  |  | 1 |
| Caucasian | 7 (17.9%) | 4 (20.0%) | 3 (15.8%) |  |
| African American/Black | 26 (66.7%) | 13 (65.0%) | 13 (68.4%) |  |
| Other | 3 (7.7%) | 1 (5.0%) | 2 (10.5%) |  |
| Did not disclose | 3 (7.7%) | 2 (10.0%) | 1 (5.3%) |  |
| Ethnicity |  |  |  | 0.394 |
| Non-Hispanic | 32 (82.1%) | 18 (90.0%) | 14 (73.7%) |  |
| Hispanic | 4 (10.3%) | 1 (5.0%) | 3 (15.8%) |  |
| Did not disclose | 3 (7.7%) | 1 (5.0%) | 2 (10.5%) |  |
| Insurance status |  |  |  | 0.731 |
| No insurance | 12 (30.8%) | 7 (35.0%) | 5 (26.3%) |  |
| Has insurance | 27 (69.2%) | 13 (65.0%) | 14 (73.7%) |  |
| Employment status |  |  |  | 0.046 |
| Full time | 7 (17.9%) | 1 (5.0%) | 6 (31.6%) |  |
| Part time | 11 (28.2%) | 7 (35.0%) | 4 (21.1%) |  |
| Unemployed | 16 (41.0%) | 11 (55.0%) | 5 (26.3%) |  |
| Student | 2 (5.1%) | 0 (0.0%) | 2 (10.5%) |  |
| Homemaker/stay-at-home parent/other | 2 (5.1%) | 0 (0.0%) | 1 (5.3%) |  |
| Did not disclose | 7 (17.9%) | 1 (5.0%) | 1 (5.3%) |  |
| Education |  |  |  | 0.28 |
| Did not graduate high school | 4 (10.3%) | 1 (5.0%) | 3 (15.8%) |  |
| High school graduate/GED | 16 (41.0%) | 11 (55.0%) | 5 (26.3%) |  |
| Technical/vocational/some college | 15 (38.5%) | 6 (30.0%) | 9 (47.4%) |  |
| College graduate | 1 (2.6%) | 2 (10.0%) | 1 (5.3%) |  |
| Graduate/professional degree | 4 (10.3%) | 0 (0.0%) | 1 (5.3%) |  |
| Household income |  |  |  | 0.286 |
| $25K > | 20 (51.3%) | 12 (60.0%) | 8 (42.1%) |  |
| $25K+ - $50K | 10 (25.6%) | 4 (20.0%) | 6 (31.6%) |  |
| $50K < | 5 (12.8%) | 1 (5.0%) | 4 (21.1%) |  |
| Did not disclose | 4 (10.3%) | 3 (15.0%) | 1 (5.3%) |  |
| FTND score | 5.8 (1.4) | 5.8 (1.5) | 5.8 (1.3) | 0.892 |

**Associations between progesterone levels and smoking-related outcomes**

**Table S2. Interaction effect of progesterone levels and sex on self-reported psychological measures.**

| **Outcome** | **Log(prog) by sex interaction**  **p value** | **Within females:**  **Effect of log(prog)**  **(95% CI)** | **P value** | **Within males:**  **Effect of log(prog)**  **(95% CI)** | **P value** | **Difference (95% CI) between slopes** |
| --- | --- | --- | --- | --- | --- | --- |
| QSU-Brief | 0.586 | -2.25 (-5.03, 0.54) | 0.114 | -1.35 (-3.57, 0.88) | 0.236 | -0.90 (-4.08, 2.27) |
| NWSC | 0.099 | -0.18 (-0.72, 0.36) | 0.516 | 0.36 (-0.29, 1.02) | 0.277 | -0.54 (-1.13, 0.04) |
| QSU* | 0.610 | 3.33 (-5.36, 12.03) | 0.452 | 1.33 (-5.14, 7.79) | 0.687 | 2.01 (-5.70, 9.71) |
| POMS: tension-anxiety | 0.096 | 0.12 (-0.30, 0.53) | 0.580 | **0.61 (0.05, 1.18)** | **0.033** | -0.50 (-1.03, 0.04) |
| POMS: depression-dejection | 0.297 | -0.03 (-0.35, 0.29) | 0.859 | 0.21 (-0.30, 0.71) | 0.421 | -0.24 (-0.68, 0.21) |
| POMS: anger-hostility | 0.853 | 0.19 (-0.38, 0.76) | 0.508 | 0.24 (-0.10, 0.59) | 0.166 | -0.05 (-0.58, 0.48) |
| POMS: fatigue | 0.187 | -0.27 (-0.66, 0.12) | 0.172 | 0.20 (-0.47, 0.86) | 0.561 | -0.47 (-1.13, 0.20) |
| POMS: vigor | 0.573 | 0.30 (-0.41, 1.02) | 0.404 | 0.05 (-0.61, 0.71) | 0.883 | 0.25 (-0.62, 1.13) |
| POMS: confusion-bewilderment | 0.494 | 0.26 (-0.00, 0.53) | 0.050 | 0.46 (-0.20, 1.13) | 0.172 | -0.20 (-0.76, 0.36) |

*Only at final scan (‘chronic’ visit); no repeated measures

QSU-Brief = Brief Questionnaire of Smoking Urges

NWSC = Nicotine Withdrawal Symptom Checklist

QSU = Questionnaire of Smoking Urges

POMS = Profile of Mood States

**Table S3. Main effects of progesterone levels and sex on self-reported psychological measures (for non-significant interactions from Table 2S).**

| **Outcome** | **Effect of log(prog)**  **(95% CI)** | **P value** | **Effect of sex**  **[ref: females]**  **(95% CI)** | **P value** |
| --- | --- | --- | --- | --- |
| QSU-Brief | -1.83 (-3.86, 0.20) | 0.078 | -0.63 (-7.52, 6.26) | 0.859 |
| NWSC | 0.06 (-0.46, 0.58) | 0.829 | 0.21 (-1.50, 1.93) | 0.808 |
| QSU* | 2.58 (-4.29, 9.47) | 0.462 | -14.95 (-34.21, 4.31) | 0.128 |
| POMS: tension-anxiety | 0.36 (-0.08, 0.79) | 0.106 | 0.59 (-0.80, 1.98) | 0.406 |
| POMS: depression-dejection | 0.08 (-0.38, 0.44) | 0.666 | -0.26 (-1.57, 1.05) | 0.700 |
| POMS: anger-hostility | 0.22 (-0.18, 0.62) | 0.289 | -0.16 (-1.47, 1.45) | 0.807 |
| POMS: fatigue | -0.06 (-0.49, 0.37) | 0.788 | 0.09 (-1.64, 1.82) | 0.918 |
| POMS: vigor | 0.18 (-0.36, 0.72) | 0.505 | 2.25 (-1.42, 5.92) | 0.230 |
| POMS: confusion-bewilderment | 0.36 (-0.05, 0.77) | 0.089 | -0.16 (-1.35, 1.03) | 0.792 |

*Only at final scan (‘chronic’ visit); no repeated measures

QSU-Brief = Brief Questionnaire of Smoking Urges

NWSC = Nicotine Withdrawal Symptom Checklist

QSU = Questionnaire of Smoking Urges

POMS = Profile of Mood States

**Table S3. Interaction effect of progesterone levels and sex on smoking session measures.**

| **Outcome at final smoking session** | **Log(prog) by sex interaction**  **p value** | **Within females:**  **Effect of log(prog)**  **(95% CI)** | **P value** | **Within males:**  **Effect of log(prog)**  **(95% CI)** | **P value** | **Difference (95% CI) between slopes** |
| --- | --- | --- | --- | --- | --- | --- |
| CO (post – pre) | 0.947 | **2.56 (0.31, 4.83)** | **0.026** | 2.33 (-0.68, 5.33) | 0.130 | 0.24 (-2.12, 2.60) |
| Number of cigarettes | 0.984 | 0.25 (-0.07, 0.57) | 0.132 | 0.24 (-0.02, 0.51) | 0.073 | 0.00 (-0.29, 0.29) |
| Volume smoked (1^st^ cigarette) | 0.382 | 145.9 (-12.0, 303.7) | 0.070 | 54.6 (-98.7, 207.9) | 0.485 | 91.3 (-109.3, 291.8) |
| Total volume smoked | 0.665 | 91.8 (-812.1, 995.9) | 0.842 | 276.1 (-137.5, 689.7) | 0.191 | -184.3 (-1018.8, 650.3) |
| NEQ1 (feel strength of nicotine) | 0.147 | -3.71 (-10.00, 2.58) | 0.248 | 0.16 (-4.60, 4.92) | 0.948 | -3.87 (-8.79, 1.05) |
| NEQ2 (feel “good” effects of nicotine) | **0.020** | **-9.54 (-14.92, -4.16)** | **<0.001** | -1.92 (-7.39, 3.56) | 0.493 | **-7.62 (-12.46, -2.80)** |
| NEQ3 (feel “bad” effects of nicotine) | 0.166 | 7.28 (-0.99, 15.55) | 0.085 | 1.72 (-5.87, 9.31) | 0.658 | 5.56 (-1.29, 12.41) |
| NEQ4 (head rush) | 0.871 | 3.02 (-6.57, 12.61) | 0.537 | 3.60 (-6.38, 13.58) | 0.480 | -0.58 (-7.65, 6.48) |

**Table S4. Main effects of progesterone levels and sex on smoking session measures (for non-significant interactions from Table 3S).**

| **Outcome at final smoking session** | **Effect of log(prog)**  **(95% CI)** | **P value** | **Effect of sex**  **[ref: females]**  **(95% CI)** | **P value** |
| --- | --- | --- | --- | --- |
| CO (post – pre) | **2.47 (0.16, 4.78)** | **0.056** | 2.15 (-2.23, 6.54) | 0.335 |
| Number of cigarettes | 0.25 (-0.01, 0.50) | 0.059 | -0.02 (-0.72, 0.69) | 0.960 |
| Volume smoked (1^st^ cigarette) | 107.9 (-13.04, 228.8) | 0.080 | -100.4 (-460.7, 260.0) | 0.585 |
| Total volume smoked | 174.3 (-442.1, 790.8) | 0.579 | -742.3 (-2363.1, 878.6) | 0.369 |
| NEQ1 (feel strength of nicotine) | -1.40 (-6.96, 4.16) | 0.621 | -0.16 (-18.23, 17.80) | 0.986 |
| NEQ2 (feel “good” effects of nicotine) | - | - | - | - |
| NEQ3 (feel “bad” effects of nicotine) | 4.87 (-2.34, 12.07) | 0.186 | 15.35 (0.01, 30.69) | 0.0498 |
| NEQ4 (head rush) | 3.42 (-5.63, 12.48) | 0.459 | 16.21 (-4.60, 37.01) | 0.127 |

**Table S5. Interaction of progesterone levels and sex on neural activation when viewing smoking cues minus neutral cues.**

| **Outcome** | **Log(prog) by sex interaction**  **p value** | **Within females:**  **Effect of log(prog)**  **(95% CI)** | **P value** | **Within males:**  **Effect of log(prog)**  **(95% CI)** | **P value** | **Difference between slopes (95% CI)** |
| --- | --- | --- | --- | --- | --- | --- |
| PCC | 0.448 | 0.00 (-0.07, 0.08) | 0.949 | 0.05 (-0.07, 0.16) | 0.430 | -0.04 (-0.16, 0.07) |
| ACC | 0.526 | 0.00 (-0.08, 0.07) | 0.910 | 0.03 (-0.09, 0.15) | 0.596 | -0.04 (-0.15, 0.07) |
| Lateral occipital cortex (left) | 0.610 | -0.02 (-0.09, 0.04) | 0.483 | 0.00 (-0.08, 0.08) | 0.992 | -0.02 (-0.11, 0.06) |
| Middle temporal gyrus (left) | 0.552 | -0.01 (-0.08, 0.06) | 0.813 | 0.02 (-0.06, 0.10) | 0.611 | -0.03 (-0.12, 0.07) |

PCC = posterior cingulate cortex; ACC = anterior cingulate cortex

**Table S6. Main effects progesterone and sex on neural activation when viewing smoking cues minus neutral cues (for non-significant interactions from Table 5S).**

| **Outcome** | **Effect of log(prog)**  **(95% CI)** | **P value** | **Effect of sex**  **[ref: females]**  **(95% CI)** | **P value** |
| --- | --- | --- | --- | --- |
| PCC | 0.02 (-0.05, 0.09) | 0.618 | **0.12 (0.001, 0.23)** | **0.048** |
| ACC | 0.01 (-0.07, 0.08) | 0.834 | 0.04 (-0.08, 0.16) | 0.524 |
| Lateral occipital cortex (left) | -0.02 (-0.07, 0.04) | 0.596 | 0.07 (-0.02, 0.16) | 0.154 |
| Middle temporal gyrus (left) | 0.00 (-0.05, 0.06) | 0.932 | 0.08 (-0.04, 0.20) | 0.179 |

PCC = posterior cingulate cortex; ACC = anterior cingulate cortex

**Associations between allopregnanolone levels and smoking-related outcomes**

**Table S7. Interaction effect of allopregnanolone levels and sex on self-reported psychological measures.**

| **Outcome** | **Log(allo) by sex interaction**  **p value** | **Within females:**  **Effect of log(allo)**  **(95% CI)** | **P value** | **Within males:**  **Effect of log(allo)**  **(95% CI)** | **P value** | **Difference between slopes (95% CI)** |
| --- | --- | --- | --- | --- | --- | --- |
| QSU-Brief | 0.422 | -1.83 (-4.13, 0.46) | 0.118 | -0.85 (-2.09, 0.40) | 0.183 | -0.99 (-3.41, 1.44) |
| NWSC | 0.181 | -0.20 (-0.68, 0.29) | 0.424 | 0.15 (-0.18, 0.49) | 0.370 | -0.35 (-0.86, 0.16) |
| QSU* | 0.682 | 5.13 (-4.85, 15.10) | 0.314 | 6.21 (-4.06, 16.48) | 0.236 | -1.08 (-6.28, 4.11) |
| POMS: tension-anxiety | 0.089 | 0.03 (-0.25, 0.30) | 0.840 | **0.34 (0.03, 0.65)** | **0.033** | -0.31 (-0.66, 0.03) |
| POMS: depression-dejection | 0.731 | 0.08 (-0.18, 0.34) | 0.542 | 0.12 (-0.10, 0.34) | 0.274 | -0.04 (-0.28, 0.20) |
| POMS: anger-hostility | 0.340 | 0.06 (-0.18, 0.29) | 0.631 | 0.17 (-0.0004, 0.35) | 0.051 | -0.12 (-0.35, 0.12) |
| POMS: fatigue | 0.081 | -0.34 (-0.73, 0.05) | 0.090 | 0.06 (-0.21, 0.34) | 0.659 | -0.40 (-0.82, 0.02) |
| POMS: vigor | 0.425 | 0.43 (-0.43, 1.30) | 0.326 | 0.08 (-0.36, 0.53) | 0.707 | 0.35 (-0.48, 1.18) |
| POMS: confusion-bewilderment | 0.211 | 0.00 (-0.29, 0.30) | 0.979 | 0.20 (-0.10, 0.50) | 0.190 | -0.19 (-0.50, 0.11) |

*Only at final scan (‘chronic’ visit); no repeated measures

QSU-Brief = Brief Questionnaire of Smoking Urges

NWSC = Nicotine Withdrawal Symptom Checklist

QSU = Questionnaire of Smoking Urges

POMS = Profile of Mood States

**Table S8. Main effects of allopregnanolone levels and sex on self-reported psychological measures (for non-significant interactions from Table S7).**

| **Outcome** | **Effect of log(allo)**  **(95% CI)** | **P value** | **Effect of sex**  **[ref: females]**  **(95% CI)** | **P value** |
| --- | --- | --- | --- | --- |
| QSU-Brief | -1.06 (-2.24, 0.13) | 0.081 | -2.78 (-9.59, 4.03) | 0.424 |
| NWSC | 0.08 (-0.23, 0.38) | 0.632 | -0.24 (-1.97, 1.49) | 0.785 |
| QSU* | 4.92 (-5.16, 15.01) | 0.339 | -19.16 (-40.48, 2.16) | 0.078 |
| POMS: tension-anxiety | **0.28 (-0.01, 0.55)** | **0.042** | 0.46 (-0.86, 1.77) | 0.499 |
| POMS: depression-dejection | 0.11 (-0.09, 0.32) | 0.285 | -0.32 (-1.49, 0.87) | 0.604 |
| POMS: anger-hostility | 0.15 (-0.01, 0.31) | 0.074 | -0.30 (-1.59, 0.98) | 0.643 |
| POMS: fatigue | -0.02 (-0.28, 0.24) | 0.883 | 0.10 (-1.57, 1.77) | 0.905 |
| POMS: vigor | 0.15 (-0.30, 0.60) | 0.517 | 2.70 (-0.87, 6.27) | 0.138 |
| POMS: confusion-bewilderment | 0.16 (-0.11, 0.43) | 0.252 | -0.43 (-1.57, 0.71) | 0.460 |

*Only at final scan (‘chronic’ visit); no repeated measures

QSU-Brief = Brief Questionnaire of Smoking Urges

NWSC = Nicotine Withdrawal Symptom Checklist

QSU = Questionnaire of Smoking Urges

POMS = Profile of Mood States

**Table S9. Interaction effect of allopregnanolone levels and sex on smoking session measures.**

| **Outcome at final smoking session** | **Log(allo) by sex interaction**  **p value** | **Within females:**  **Effect of log(allo)**  **(95% CI)** | **P value** | **Within males:**  **Effect of log(allo)**  **(95% CI)** | **P value** | **Difference between slopes (95% CI)** |
| --- | --- | --- | --- | --- | --- | --- |
| CO (post – pre) | 0.999 | 1.25 (-1.14, 3.64) | 0.306 | 1.25 (-0.60, 3.10) | 0.186 | 0.00 (-1.80, 1.80) |
| Number of cigarettes | 0.991 | 0.25 (-0.10, 0.59) | 0.159 | 0.24 (-0.04, 0.53) | 0.091 | 0.00 (-0.19, 0.19) |
| Volume smoked (1^st^ cigarette) | 0.428 | 84.3 (-154.6, 323.2) | 0.489 | 29.8 (-179.8, 239.4) | 0.781 | 54.5 (-80.8, 189.8) |
| Total volume smoked | 0.894 | 489.0 (-241.0, 1219.0) | 0.189 | **526.5 (93.2, 959.9)** | **0.017** | -37.5 (-573.9, 498.9) |
| NEQ1 (feel strength of nicotine) | 0.387 | -1.61 (-9.12, 5.90) | 0.675 | 0.40 (-4.65, 5.45) | 0.877 | -2.01 (-6.41, 2.40) |
| NEQ2 (feel “good” effects of nicotine) | 0.183 | -1.47 (-10.11, 7.17) | 0.739 | 2.70 (-2.72, 8.13) | 0.328 | -4.18 (-9.70, 1.34) |
| NEQ3 (feel “bad” effects of nicotine) | 0.105 | 1.28 (-6.25, 8.80) | 0.739 | -3.21 (-9.75, 3.33) | 0.336 | 4.48 (-0.40, 9.37) |
| NEQ4 (head rush) | 0.389 | -7.52 (-17.03, 1.99) | 0.121 | -4.79 (-13.05, 3.47) | 0.256 | -2.73 (-8.89, 3.44) |

**Table S10. Main effects of allopregnanolone levels and sex on smoking session measures (for non-significant interactions from Table S9).**

| **Outcome** | **Effect of log(allo)**  **(95% CI)** | **P value** | **Effect of sex**  **[ref: females]**  **(95% CI)** | **P value** |
| --- | --- | --- | --- | --- |
| CO (post – pre) | 1.25 (-0.61, 3.11) | 0.189 | 1.16 (-3.44, 5.76) | 0.621 |
| Number of cigarettes | 0.25 (-0.04, 0.53) | 0.087 | -0.11 (-0.78, 0.57) | 0.759 |
| Volume smoked (1^st^ cigarette) | 35.1 (-170.2, 240.4) | 0.738 | -149.9 (-540.4, 240.6) | 0.452 |
| Total volume smoked | **524.1 (81.8, 966.4)** | **0.020** | -801.9 (-2282.2, 678.5) | 0.288 |
| NEQ1 (feel strength of nicotine) | 1.40 (-3.67, 6.46) | 0.589 | 0.80 (-16.62, 18.22) | 0.929 |
| NEQ2 (feel “good” effects of nicotine) | 4.04 (-1.54, 9.61) | 0.156 | -8.89 (-24.72, 7.95) | 0.301 |
| NEQ3 (feel “bad” effects of nicotine) | -3.23 (-9.61, 3.15) | 0.321 | 12.33 (-3.66, 28.31) | 0.131 |
| NEQ4 (head rush) | -4.03 (-12.34, 4.29) | 0.343 | 13.87 (-6.37, 34.12) | 0.179 |

**Table S11. Interaction of allopregnanolone levels and sex on neural activation when viewing smoking cues minus neutral cues.**

| **Outcome** | **Log(allo) by sex interaction**  **p value** | **Within females:**  **Effect of log(allo)**  **(95% CI)** | **P value** | **Within males:**  **Effect of log(allo)**  **(95% CI)** | **P value** | **Difference between slopes (95% CI)** |
| --- | --- | --- | --- | --- | --- | --- |
| PCC | 0.953 | -0.01 (-0.08, 0.06) | 0.809 | -0.01 (-0.08, 0.06) | 0.771 | 0.00 (-0.06, 0.07) |
| ACC | 0.697 | -0.03 (-0.09, 0.03) | 0.282 | -0.02 (-0.07, 0.03) | 0.389 | -0.01 (-0.07, 0.05) |
| Lateral occipital cortex (left) | 0.735 | -0.04 (-0.10, 0.01) | 0.147 | -0.03 (-0.08, 0.01) | 0.165 | -0.01 (-0.06, 0.04) |
| Middle temporal gyrus (left) | 0.976 | 0.00 (-0.06, 0.05) | 0.884 | 0.00 (-0.06, 0.05) | 0.859 | 0.00 (-0.05, 0.05) |

PCC = posterior cingulate cortex; ACC = anterior cingulate cortex

**Table S12. Main effects allopregnanolone levels and sex on neural activation when viewing smoking cues minus neutral cues (for non-significant interactions from Table 11S).**

| **Outcome** | **Effect of log(allo)**  **(95% CI)** | **P value** | **Effect of sex**  **[ref: females]**  **(95% CI)** | **P value** |
| --- | --- | --- | --- | --- |
| PCC | -0.01 (-0.08, 0.06) | 0.767 | 0.13 (-0.004, 0.26) | 0.057 |
| ACC | -0.02 (-0.07, 0.03) | 0.356 | 0.07 (-0.07, 0.20) | 0.343 |
| Lateral occipital cortex (left) | -0.03 (-0.08, 0.01) | 0.149 | 0.10 (-0.01, 0.22) | 0.087 |
| Middle temporal gyrus (left) | -0.005 (-0.05, 0.05) | 0.856 | 0.08 (-0.03, 0.20) | 0.164 |

PCC = posterior cingulate cortex; ACC = anterior cingulate cortex

**Table S13. Functionally defined regions of interest (ROIs) included in analysis, originally identified for the smoking minus neutral contrast at the baseline session with a threshold of 200 voxels and voxel-wise correction of *p* = 0.05.**

| **Functional**  **Region of Interest** | **Cluster Index** | **Voxels** | **P value** | **MAX (Z- Score)** | **MAX X (mm)** | **MAX Y (mm)** | **MAX Z (mm)** |
| --- | --- | --- | --- | --- | --- | --- | --- |
| PCC | 19 | 1358 | 2.43E-12 | 6.82 | 2 | -48 | 26 |
| ACC | 18 | 888 | 6.52E-10 | 6.17 | -2 | 38 | -2 |
| Lateral Occipital Cortex, Left | 17 | 720 | 6.05E-09 | 6.03 | -48 | -70 | 28 |
| Middle Temporal Gyrus, Left | 16 | 280 | 6.26E-06 | 5.99 | -60 | -6 | -24 |

PCC = posterior cingulate cortex; ACC = anterior cingulate cortex

**References:**

1. Heatherton TF, Kozlowski LT, Frecker RC, Fagerstrom K-O (1991): The Fagerstrom Test for Nicotine Dependence: a revision of the Fagerstrom Tolerance Questionnaire. *Addiction* 86: 1119–1127.

2. Smith SM (2002): Fast robust automated brain extraction. *Hum Brain Mapp* 17: 143–155.

3. Jenkinson M, Smith S (2001): A global optimisation method for robust affine registration of brain images. *Medical Image Analysis* 5: 143–156.

4. Jenkinson M, Bannister P, Brady M, Smith S (2002): Improved optimization for the robust and accurate linear registration and motion correction of brain images. *Neuroimage* 17: 825–841.

5. Woolrich MW, Ripley BD, Brady M, Smith SM (2001): Temporal autocorrelation in univariate linear modeling of FMRI data. *Neuroimage* 14: 1370–1386.
